# Abdominal Cocoon Syndrome: A Laparoscopic Approach

**DOI:** 10.7759/cureus.16787

**Published:** 2021-07-31

**Authors:** Waqas Aziz, Yashfeen Malik, Shahan Haseeb, Rida T Mirza, Sameen Aamer

**Affiliations:** 1 General Surgery, Shifa International Hospital Islamabad, Islamabad, PAK; 2 Internal Medicine, Shifa International Hospital Islamabad, Islamabad, PAK

**Keywords:** abdominal cocoon syndrome, laparoscopic technique, intestinal obstruction, sclerosing encapsulating peritonitis, acute abdomen

## Abstract

Sclerosing encapsulating peritonitis, or abdominal cocoon syndrome (ACS), is a rare cause of intestinal obstruction in which the small bowel is encapsulated by a fibro-collagenous membrane. We present the case of a 29-year-old male who presented to us with acute intestinal obstruction. The imaging performed suggested the presence of ACS. The patient underwent laparoscopic adhesiolysis and the small bowel was released. In cases of recurrent small bowel obstruction, a high index of suspicion is required for the diagnosis of ACS. Computed tomography can be a useful imaging modality, and surgery remains the mainstay of treatment.

## Introduction

Sclerosing encapsulating peritonitis (SEP) is a rare cause of intestinal obstruction where the small bowel is encased in a fibro-collagenous membrane. Usually occurring in young females, it is a rare condition categorized into two classes, namely, primary and secondary. If no etiology is identified, the entity is labeled as primary or idiopathic SEP, also known as the abdominal cocoon syndrome (ACS). Known causes of secondary SEP include peritoneal dialysis, pelvic inflammatory disease, disseminated tuberculosis, and sarcoidosis [1]. SEP has also been known to occur in patients with a history of liver transplants [2]. It is characterized by a thick grayish-white fibrotic membrane that partially or completely encases the small bowel in a concertina-like manner. It may extend into other organs such as the large bowel, liver, or stomach, and usually presents with symptoms of recurrent intestinal obstructions [3].

Here, we describe the case of a 29-year-old male who presented with the signs and symptoms of acute intestinal obstruction. A combination of radiological studies and diagnostic laparoscopy was used to reach a diagnosis of ACS.

## Case presentation

A 29-year-old gentleman presented to the emergency department with complaints of abdominal pain, vomiting, and absolute constipation for the past day. The pain was focused on the epigastric and lumbar regions and was temporarily relieved with over-the-counter analgesics. The vomiting was nonbilious in nature and associated with oral intake. He reported suffering from similar episodes in the past with multiple hospital admissions, with the last episode being three years ago, which resolved on conservative management. The patient had no history of tuberculosis or any positive contacts. There was no history of previous surgeries.

Upon examination, the patient was vitally stable and the abdomen was mildly distended with tenderness in the epigastric region. The patient had hyperactive bowel sounds and the per rectal examination was not significant. Baseline investigations revealed a white cell count of 14,400 U/L (range: 4,000-11,000 U/L). His electrolytes and all pertinent labs were within normal limits.

**Figure 1:**
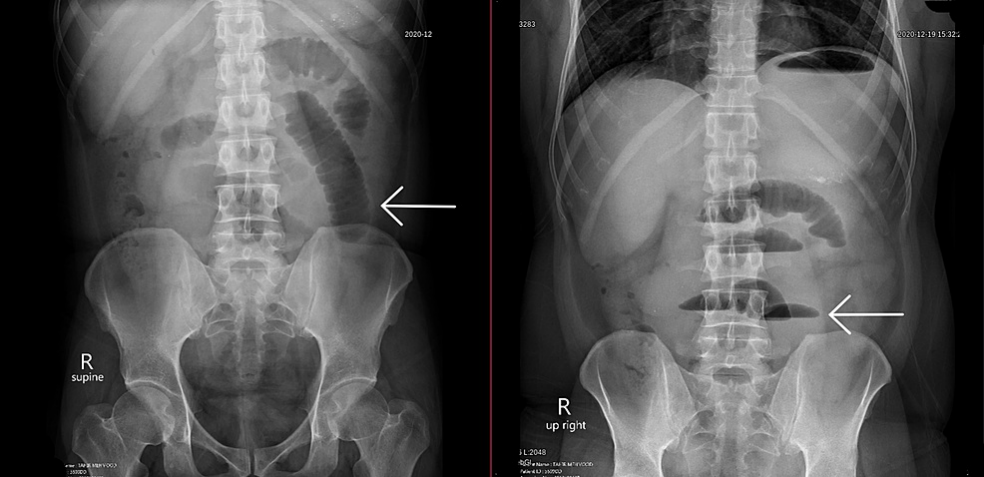
Abdominal X-ray. X-ray demonstrates gas-filled small bowel loops (Left) with numerous air-fluid levels (Right), suggesting small bowel obstruction.

An abdominal X-ray was performed (Figure 1) which revealed mildly dilated gas-filled small bowel loops, few large air-fluid levels, with the largest at 6.6 cm, paucity of gas in the rectum, and no evidence of pneumoperitoneum. The X-ray was suggestive of obstruction, without a clear etiology.

**Figure 2:**
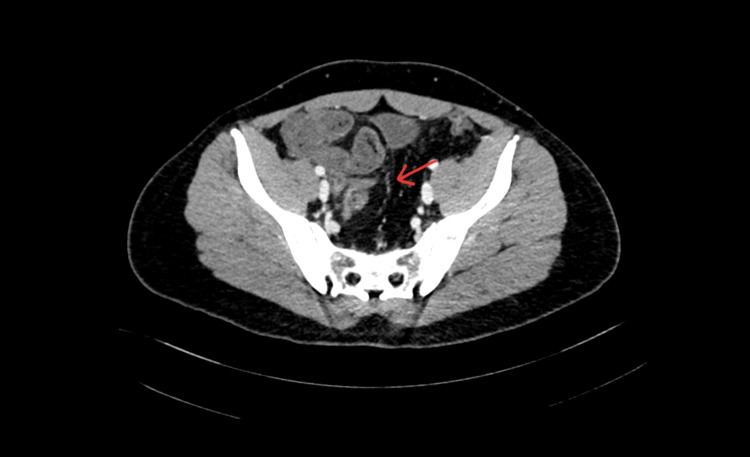
CT of the abdomen and pelvis. A thin membrane surrounding the small bowel is visible (arrow). CT: computed tomography

A computerized tomography (CT) scan of the abdomen and pelvis (Figure 2) was performed which showed mildly distended small bowel loops with a transition point at the ileocecal junction and associated mild mesenteric congestive changes suggesting small bowel obstruction with collapsed cecum and colon. The bowel loops in the right hemipelvis appeared clumped with the surrounding thin membrane. The CT was concerning for abdominal cocoon.

Conservative management was not attempted in this admission. A diagnostic laparoscopy to confirm the preoperative diagnosis was planned and carried out. Peroperatively, about 35 to 40 cm of small bowel was found to be encased in a thin grayish-white membrane till the distal ileum. A long, inflamed appendix was also visualized. A peroperative diagnosis of abdominal cocoon was confirmed.

Laparoscopically, adhesiolysis was done and the entire small bowel was carefully released from the membrane with blunt and fine dissection using endoscopic scissors (Video 1).

**Video 1:** Laparoscopic adhesiolysis. Dissection of the encapsulating membrane was performed using endoscopic scissors.

The patient had an uneventful postoperative course. He was started on a liquid diet on postoperative day one which was progressed to a soft diet by postoperative day two. The patient’s constipation was resolved and he was discharged from the hospital on postoperative day three.

## Discussion

Primary or idiopathic SEP, also known as ACS, was first described in 1978. A rare condition known to usually affect adolescent girls, it also occurs rarely in older males. It is characterized by a thick fibro-collagenous membrane encasing the small bowel. ACS is categorized into three main types based on the extent of encasing membrane and its involvement of various organs [4]. In type 1, only a part of the small intestine is covered. In type 2, the small intestine is covered completely. In type 3, the fibro-collagenous membrane extends to cover various organs such as the stomach, appendix, cecum, ascending colon, and/or ovaries.

Secondary SEP, where an identifiable etiology is present, is more common and associated with peritoneal dialysis, peritoneal venous shunting, beta-blocker use, penetrating abdominal injuries, tuberculosis, sarcoidosis, as well as liver transplantation [1,2,5].

The primary form of the etiology, although more unusual, presents with signs and symptoms of subacute intestinal obstruction. Patients usually suffer from recurrent episodes of generalized abdominal cramps, vomiting, and distension which may resolve with conservative management.

Preoperatively, the entity is difficult to diagnose due to a lack of early clinical manifestations and specific signs. Careful analysis of imaging can be helpful and can save the patient from gut resection. An abdominal X-ray may show signs of acute obstruction with air-fluid levels and dilated intestines. It may also identify a calcified membrane around the small bowel. CT is by far the most useful imaging technique, and recognition of dilated small gut in the central abdomen with an encapsulating membrane around can be diagnostic [2,6-8]. CT may also show enhancement of peritoneum, ascites, thickening of bowel walls, or reactive adenopathy among other nonspecific signs. In addition, CT can identify the extent of bowel involved and other visceral involvement or complications. Another preoperative diagnostic tool is a barium follow-through which shows a “cauliflower” sign representative of clumping of the small gut encased in a membrane. However, a definitive diagnosis of the entity is made peroperatively. An exploratory laparotomy or diagnostic laparoscopy, as in our case, followed by the release of the small bowel by removal of the membrane is diagnostic as well as therapeutic [9]. There is no indication of gut resection unless ischemia or gangrene is detected. Histopathology of the peroperative samples of the membrane shows fibrous tissue with mild inflammation, as in our case.

Despite its rarity, the condition is highly treatable with an excellent prognosis and surgical intervention with removal of the sac and adhesiolysis leads to a full recovery. Enterectomy is only performed for patients with intestinal necrosis, but anastomosis of the intestinal loops is not recommended because it may cause a high percentage of surgical complications, such as intestinal fistula. If resection needs to be performed, a stoma is highly recommended, especially in patients who have a gastrointestinal perforation [6].

Complications include infections, adhesions, enterocutaneous fistulae, and low complication rates for ACS compared to secondary SEP arising from peritoneal dialysis [7].

## Conclusions

ACS is a rare condition that presents as intestinal obstruction due to the small intestine being wrapped in a fibro-collagenous membrane. Although it is diagnosed peroperatively, with modern imaging techniques, it is possible to visualize the membrane preoperatively and plan accordingly. Differential for any patient presenting with recurrent obstruction should include ACS. Surgery is the mainstay of treatment and complete resolution of symptoms is achieved with a low rate of complications.
